# Strategies for preventing group B streptococcal infections in newborns: a nation-wide survey of Italian policies

**DOI:** 10.1186/s13052-017-0409-1

**Published:** 2017-11-02

**Authors:** Chryssoula Tzialla, Alberto Berardi, Claudio Farina, Pierangelo Clerici, Alessandro Borghesi, Elsa Viora, Paolo Scollo, Mauro Stronati, Task Force for group B streptococcal infections for the Italian Society of Neonatology, G. Stival, M. A. Barbaglia, A. Guala, E. Giunta, L. Parola, M. R. Grossignani, P. Perri, L. Tubaldi, G. Alletto, S. Daidone, V. Flacco, C. Dani, A. Sterpa, G. Rapisardi, M. R. Elicio, G. Faldella, M. G. Capretti, H. Messner, M. Bandiera, C. Achille, A. Azzali, G. Montrasio, S. Mariani, G. Galvagno, E. Giacosa, F. de Angelis, M. Spandrio, A. Serra, F. Garofalo, A. Perona, F. Porcelli, F. Ferrero, S. De Franco, P. Paollilo, S. Picone, R. Besana, T. Varisco, M. Farina, L. Memo, G. Nicolini, D. Lietti, G. Di Chiara, A. Rottoli, T. Bonabitacola, E. Cortis, E. Neri, S. Martinelli, L. Ilardi, G. F. Rondanini, P. Calzi, A. Gatta, P. A. Quntadamo, M. Ivaldi, L. Terenzani, N. Di Lascio, M. D. Travaglio, G. Vetrano, G. Furcolo, V. Vitacco, C. Intini, M. Frigerio, P. Stroppiana, G. Policicchio, P. Mesirca, P. Gianino, E. Audenio, R. Paludetto, F. Raimondi, A. Pugliese, L. Valentino, N. Nosari, G. Marchesano, G. Chirico, R. Bellù, M. Menchini, A. Poletti, T. Vacchiano e, L. Pinto, D. Perri e, R. Coppola, R. Perini, A. Vetrella, G. De Luca, G. Lista, F. Cavigioli, A. Bettinelli, E. Massironi, C. Franco, L. Bernardo, S. Poli, M. Palladini, V. Tota, F. Spadavecchia, G. V. Zuccotti, L. Pogliani, G. Bracaglia, A. L. Mancini, F. Zocco, G. Iozzia, A. Auriemma, M. Teani, G. Mangilli, A. M. Tempra, L. Di Terlizi, R. Bottino, C. Salvi, V. Fortunato, R. Musaico, G. Gargantini, G. Carrera, R. Magaldi, L. Taurino, A. M. D’Onofrio, E. Buffone, A. Tempera, M. Agosti, P. Garzia, F. Mosca, L. Pugni, P. Tagliabue, C. Colombo, M. Demi, G. Picco, A. Carlucci, G. Zorzi, D. Padula, M. L. Cardone, G. Buonocore, M. C. Muraca, A. Boldrini, M. Ciantelli, M. Lanari, L. Serra, L. Felici, G. Banderalli, C. Brambilla, A. Dall’Agnola, E. Viviani, M. C. Zonca, G. Licardi, A. Chiara, G. Ancora, I. Papa, P. Gancia, G. Pomero, A. Deloglu, P. Villani, P. Mussini, E. Canidio, D. Migliavacca, S. Di Fabio, I. Cipollone, G. Biasucci, P. Rubbi, M. Piepoli, N. Guastaferro, F. Infriccioli, E. Bertino, C. Perathoner, S. Parmigiani, G. Suriano, C. Ianniello, A. Biasini, M. Azzalli, G. Timpani, S. Barresi, G. Caoci, R. Ciccotti, M. De Curtis, F. Natale, M. Finocchi, C. Haass, F. Milillo, S. Lucieri, E. Guercio, S. A. Canepa, G. Scozia, R. Antonucci, O. Limongelli, S. Macciò, F. Mongelli, F. Colonna, D. Dragovic, M. T. Calipa, A. Cohen, L. Moresco, Italian Society of Obstetricians and Gynecologists, R. La Spina, R. Ruggeri, A. Luehwink, M. Brattoli, A. Fedi, L. Lacchi, G. Ettore, E. Pappalardo, G. Conoscenti, B. Zeni, D. Spellecchia, L. Favretti, L. Spagna, S. Zaglio, D. Bresciani, A. Bandini, R. Mancini, P. Mustoni, D. Dodero, M. Grimaldi, M. Di Mario, P. Migliorini, A. Kemeny, P. S. Anastasio, T. Riccardi, T. Maggino, G. Cerri, P. Puggina, A. M. Marconi, S. Morgia, G. Bellia, M. R. D’Anna, M. Catania, A. Bacchi Modena, L. Franchi, N. Calonaci, S. Schettini, R. Paradiso, P. Saccucci, M. Ioppi, M. Zorzi, G. Stellin, F. Patacchiola, L. Carrata, D. Bassini, L. San Marco, T. Todros, C. Tibadi, M. Liborio, Italian Association of Clinical Microbiologists, R. Laricchia, L. Tauro, F. Ferrara, C. Nuara, E. Ghiraldi, F. Molinari, A. Comessatti, A. Rocchetti, L. Di Matteo, V. Miconi, P. Calvi, A. Pernigotti, F. Fabozzi, G. Micca, G. Monticone, M. Sarti, G. Da Rin, M. Zoppelletto, E. Modolo, M. P. Landini, G. Furlini, E. Galluppi, E. Pagani, R. Aschbacher, P. Innocenti, N. Bresolin, M. E. Raggi, C. Bonfanti, M. De Francesco, P. Santer, A. Griessmaier, D. De Francesco, A. Pirali, C. Prasciolu, F. Usai, G. Cuzzone, M. Scutellà, P. Tramacere, D. Fossati, G. Piaserico, G. Bordignon, A. Sciacca, F. Di Vincenzo, A. Imbriani, D. Melotti, G. Catanoso, I. Rivetti, G. Neri, R. Bruno, L. Bacelle, P. Sartore, G. Giana, E. Sala, C. Giraldi, P. Cavalcanti, M. Perugini, A. Perugini, C. Ginardi, D. Maritano, A. Ferrini, A. Bonettini, A. Avanzini, S. Gasperoni, B. Pieretti, E. Montanari, C. Carillo, M. R. Rossi, A. Laureti, M. L. Baldoni, D. Serra, G. Melioli, R. Bandettini, F. Oneto, R. Colla, S. Storchi Incerti, G. Catanoso, F. Lanzini, P. Pauri, E. Tili, R. A. Leone, G. Verdastro, M. Megha, F. Luzzaro, A. Conti, L. Busulini, P. Mirri, R. Diodati, C. Vettori, S. Pittalis, A. Anesi, A. Fiore, L. Goglia, E. Vitullo, A. Sinno, S. Platzgummer, C. Spitaler, M. C. Trabucchi, M. Besozzi, E. Cesana, G. Inghilleri, S. Grosso, R. D’Angelo, E. Fogato, F. Lavarda, G. Ortisi, M. Clementi, P. Cichero, F. Rumpianesi, C. Venturelli, F. Mortillaro, S. Daffara, M. R. Catania, D. Iula, S. Andreoni, A. Politi, C. Agostinelli, C. Paparella, D. Capozzi, P. Notaris, F. Bistoni, A. Mencacci, M. Valentini, A. Filippetti, M. Confalonieri, O. Novarese, F. Bonini, D. Salamone, A. Camporese, R. De Rosa, P. Casprini, R. Degl’Innocenti, R. Giordano, M. T. Allù, D. Zanella, M. Malandrino, M. Tronci, M. Valmarin, G. Leonetti, S. Falco, M. Meledandri, M. Ballardini, A. Spanò, M. C. Cava, M. T. Mascellino, M. Schinella, P. Gualdi, E. Casari, N. Scattolo, C. Motta, C. Perfetti, M. Bassano, G. Cera, P. Iafisco, I. Mura, A. Palmieri, M. Migliardi, M. Ferlini, G. Grandi, F. Giardini, F. Albano, M. Latino, M. P. Ferrero, L. Bellizia, M. Russolo, S. Russolo, A. Pesenti, M. A. Fasano, S. Previato, O. Radillo, M. Busetti, P. Ferrari, V. Siderini, L. Puzzolante, C. Scarparo, A. Arzese, N. Cappuccia, L. Lodolo, L. Delledonne, A. Gramoni, V. Maiolo, A. Gheller, S. Spadaro, M. Balzaretti

**Affiliations:** 1 Neonatologia, Patologia Neonatale e Terapia Intensiva Neonatale, Fondazione IRCCS Policlinico, Viale Golgi 11, 27100 Pavia, Italy; 2 Neonatal Intensive Care Unit, Policlinico University Hospital, Modena, Italy; 3 USC Microbiologia e Virologia ASST ‘Papa Giovanni XXIII’, Bergamo, Italy; 4 U.O. Microbiologia, A.S.S.T Ovest Milanese, Legnano, Italy; 5 SSD Ecografia Ostetrica-Ginecologica e Diagnosi Prenatale, Dipartimento di Ginecologia e Ostetricia, Azienda Ospedaliera Universitaria “Città della Salute e della Scienza”, Torino, Italy; 6 Dipartimento Materno Infantile, UOC di Ostetricia e Ginecologia Azienda Ospedaliera Cannizzaro, Catania, Italy

**Keywords:** Group B streptococcus, GBS, Survey, Infection, Neonate, Newborn infant

## Abstract

**Background:**

There are no Italian data regarding the strategies for preventing neonatal group B streptococcal (GBS) infection. We conducted a national survey in order to explore obstetrical, neonatal and microbiological practices for the GBS prevention.

**Methods:**

Three distinct questionnaires were sent to obstetricians, neonatologists and microbiologists. Questionnaires included data on prenatal GBS screening, maternal risk factors, intrapartum antibiotic prophylaxis, microbiological information concerning specimen processing and GBS antimicrobial susceptibility.

**Results:**

All respondent obstetrical units used the culture-based screening approach to identify women who should receive intrapartum antibiotic prophylaxis, and more than half of the microbiological laboratories (58%) reported using specimen processing consistent with CDC guidelines. Most neonatal units (89 out of 107, 82%) reported using protocols for preventing GBS early-onset sepsis consistent with CDC guidelines.

**Conclusions:**

The screening-based strategy is largely prevalent in Italy, and most protocols for preventing GBS early-onset sepsis are consistent with CDC guidelines. However, we found discrepancies in practices among centers that may reflect the lack of Italian guidelines issued by public health organizations.

**Electronic supplementary material:**

The online version of this article (10.1186/s13052-017-0409-1) contains supplementary material, which is available to authorized users.

## Background

Preventive strategies reduce cases of group B streptococcus early-onset sepsis. There are no previous Italian data on strategies for preventing group B streptococcus early-onset sepsis, although partial, area-based information has been recently reported from 2 Italian regions.

This study provides an overview of Italian strategies for preventing group B streptococcus early-onset sepsis and evaluate the attitudes and practices of obstetrical and neonatal units as well as microbiological laboratories. It shows that a screening-based strategy is widespread throughout the national territory despite the low response rate. However, the discrepancies that have emerged in practices among prenatal and neonatal providers as well as among laboratorians may reflect the lack of national guidelines issued by public health organizations. Future interventions should rely on further epidemiological data.

## Introduction

Since 1970, Group B Streptococcus (GBS) has emerged as the predominant cause of early-onset sepsis (EOS, sepsis occurring within the first week of life [1]). Over the past 10 years, due to the widespread of intrapartum antibiotic prophylaxis (IAP), a significant reduction in the incidence rates of the GBS-EOS has been achieved [1]. Nevertheless, GBS remains a leading cause of neonatal morbidity and mortality in industrialized countries, and causes over one-third of cases of invasive EOS in the US [1–5].

There is still controversy over how to identify women who should receive IAP. Currently, there are two main approaches: under the culture-based approach, recommended in US [5], IAP is offered to women with GBS bacteriuria during the current pregnancy, or to women who have previously delivered an infant with invasive GBS disease or who are identified as GBS colonized at the vaginal-rectal site in late pregnancy (35–37 weeks’ gestation). Under the risk-based approach (presence at the onset of labour of one of the following: previous infant with invasive GBS disease, GBS bacteriuria during any semester of current pregnancy, delivery at < 37 weeks’s gestation, amniotic membrane rupture ≥ 18 h, *intrapartum* temperature ≥ 38.0 °C), recommended in some European countries, IAP is given to women with risk factors for EOS [3]. However, a Centers for Disease Control and Prevention (CDC) sponsored population-based, retrospective cohort study demonstrated that the screening-based approach was at least 50% more effective than the risk-based strategy [5]. After the release of the CDC guidelines in 1996, and their subsequent update in 2002 and 2010 [3, 4], the incidence of GBS-EOS has declined in the US from 1.7 cases/1000 live births to 0.34–0.37 cases/1000 live births [5]. The case fatality ratio has also declined, over the past 25 years, from 25 to 50% to 4–6% [5–7]. Furthermore, the meningeal invasion accompanying GBS-EOS has reduced from 25% in the 1980s to the current 4% of cases [6].

In 1996, the Italian Society of Perinatal Medicine issued recommendations for the prevention of GBS-EOS based on universal vaginal screening [8]. However, these recommendations have not been updated since then. In March 2012, in order to promote the knowledge and endorse the use of the 2010 guidelines from CDC, the Italian Society of Neonatology posted a translated version in its website.

There are currently no data available regarding policies for preventing GBS-EOS in Italian hospitals. Therefore, we conducted a nationwide questionnaire survey in order to describe the GBS-EOS preventive practices in maternity and neonatal units and in microbiology laboratories.

## Methods

In July 2012, questionnaires were sent to the heads of all Italian maternity and neonatal units and the directors of the microbiology laboratories, and to all members of the Italian Society of Obstetricians and Gynecologists, the Italian Society of Neonatology and the Italian Association of Clinical Microbiologists. Three different questionnaires, approved by their respective scientific societies, were sent to the head of each of the obstetric and neonatal units, and microbiological laboratories (online supplement). The questionnaires were sent in electronic format, were accompanied by a cover letter asking to report the policies in use to prevent GBS-EOS, and feedback was sought with reminders.

Questionnaires were designed to gather information about maternal, neonatal and microbiological aspects of prevention of GBS-EOS and included demographic information, data on maternal prenatal or *intrapartum* GBS screening, data on IAP, risk factors for invasive GBS-EOS (e.g. prolonged rupture of membranes, preterm delivery, maternal fever, GBS bacteriuria during pregnancy, previous infant with GBS disease), hypothetical scenarios for managing at risk babies and microbiological data on specimen processing and resistant GBS strains. The neonatal questionnaire also asked to report retrospectively the number of live births from January 1st to December 31st, 2011, and the number of GBS isolates from blood and/or cerebrospinal fluid (CSF) in each unit.

Statistical analysis was performed using the Chi-squared test and Mann–Whitney test for independent samples, as appropriate. A *p* value <0.05 was considered statistically significant.

## Results

Responses from each professional category are presented, according to the respective geographical areas, in Table 1 and Additional file 1: Table S1. Questionnaires were returned from 34 out of 493 obstetrical units (7%), from 107 out of 493 neonatal units (22%), and from 101 out of 338 microbiological laboratories (30%).

**Table 1:** Respondents divided by geographic area

	Maternity units (*n*)	Responses from neonatologists *n* (%)	Responses from obstetricians *n* (%)	Microbiological Laboratories (*n*)	Responses from microbiologists *n* (%)
North Italy	193	63 (32.64)	18 (9.3)	151	68 (45)
Central Italy	101	15 (14.8)	4 (3.9)	70	17 (24.3)
South Italy	199	29 (14.6)	12 (6)	117	16 (13.7)
TOTAL	493	107 (21.7)	34 (6.9)	338	101 (29.8)

### Obstetrical data

All respondent centers used the culture-based screening approach to identify women who should receive IAP. Nearly all respondents (88%) reported specimen collection for screening of GBS colonization both at the lower vagina and ano-rectal sites. When asked about routine timing for collection of samples, 91% of respondents reported using collection at 35–37 weeks, according to the CDC guidelines. No units reported using routinely bedside rapid molecular test for *intrapartum* screening of pregnant women. Collection of urine cultures in asymptomatic patients was extremely variable among units.

Most respondents administered ampicillin as their first-line agent for IAP. Ampicillin was administered every 4 h until delivery (24 units) or every 6 h (3 units) or was not reported (7 units). For patients reporting a history of allergy (without anaphylaxis) after exposure to penicillin, the following antimicrobials were most commonly given: clindamycin (13 units), macrolides (13 units) - including clarithromycin (4 units), erythromycin (5 units), and azithromycin (2 units) - cefazolin/cephalosporin (6 units), gentamycin (2 units) or vancomycin (1 unit). Of note, only 4 units (12%) reported to test antimicrobial susceptibility for penicillin-allergic women.

### Neonatal data

Italian live births during 2011 were 554.428 (of which 261.083 in the North; 120.202 in the Centre; and 173.143 in the South of Italy). There were 171.390 live births in respondent units during 2011, with wide differences in live births among centers. The total number of blood and CSF cultures collected in neonates in each centers, as well as indications for performing a lumbar puncture is unavailable; however, positive blood cultures were 86 and positive CSF cultures were 13. The incidence rates of GBS sepsis and meningitis were therefore 0.50/1000 and 0.075 per 1000 live births, respectively. Incidence rates of GBS sepsis were significantly lower in Northern (0.36/1000 live births) compared to Central (0.77/1000 live births, *p* = 0.017) and Southern Italy (0.70/1000 live births, *p* = 0.003) (Table 2).

**Table 2:** Incidence of positive blood and CSF culture stratified by geographic area

	North Italy	Central Italy	South Italy
LB^a^ per respondent units	108.725	30.352	32.313
Positive blood cultures (*n*)	40	21	25
Incidence per 1000 LB^a^	0.36	0.77	0.70
Positive CSF cultures (*n*)	5	6	2
Incidence per 1000 LB^a^	0.045	0.22	0.056

^a^ *LB* live births

One hundred and six out of 107 neonatal units had protocols for GBS-EOS prevention and 89 (82%) established their policies based on the CDC guidelines. Preventive policies were adopted since 2000 in all units.

Previous infant with GBS sepsis, maternal GBS colonization, GBS bacteriuria during the current pregnancy, prolonged membrane rupture ≥ 18 h, chorioamnionitis, *intrapartum* fever and prematurity (< 37 weeks) were considered risk factors. Additional but less common (8%) risk factors included the following: young age and African race of mother, persistent fetal tachycardia, amniotic fluid stained with meconium or malodorous meconium, neonatal colonization at mucous sites (auricular and pharyngeal).

The management of neonates at risk for GBS EOS in different clinical scenarios is summarized in Table 3.

**Table 3:** Strategies for managing asymptomatic babies at risk for EOS

Scenarios	Observation ≥ 48 h	Observation ≥ 48 h + laboratory evaluation	Observation ≥ 48 h + laboratory evaluation + antibiotic therapy
Chorioamnionitis ± IAP, *n* ^a^	5 (5%)	38 (35%)	64 (60%)
Term neonate			
Adequate IAP, *n*	94 (88%)	12 (11%)	1 (1%)
Inadequate IAP, *n*	26 (24%)	49 (46%)	32 (30%)
Preterm neonate			
Adequate IAP, *n*	61 (57%)	44 (41%)	2 (2%)
Inadequate IAP, *n*	9 (8.5%)	52 (48.5%)	46 (43%)

^a^ *IAP* intrapartum antibiotic prophylaxis, *n* = number (and percentage) of centers with a given strategies

In ninety-four units (88%), sepsis workup was not indicated for asymptomatic full-term neonates born to a GBS colonized mother exposed to adequate IAP (penicillin, ampicillin or cefazolin administered more than 4 h prior to delivery), and only observation for ≥ 48 h was recommended. However, 81 (75.7%) respondents reported laboratory evaluation and/or antimicrobial administration for asymptomatic full-term neonates exposed to inadequate IAP (less than 4 h prior to delivery).

Approximately 40% of units reported blood testing (i.e. white blood cell count, [WBC], blood culture, C reactive protein) for asymptomatic preterm neonates exposed to adequate IAP. Moreover, nearly all respondents (98; 91.5%) reported laboratory evaluation and/or antimicrobial treatment for preterm neonates born to mothers carrying GBS exposed to inadequate IAP. Empirical antimicrobial treatment was given to chorioamnionitis exposed neonates (60% of cases) regardless of gestational age or the duration of IAP; empirical antimicrobial treatment was also given to preterm neonates exposed to inadequate IAP (43% of cases).

### Microbiological data

The stratification of data according to geographical distribution of respondents is reported in Table 4.

**Table 4:** Distribution of swabs during a 3 years period divided by geographic area

	North Italy	Central Italy	South Italy
Vaginal swabs	36.295	9.673	9.883
Vaginal-rectal swabs	71.646	15.234	7.559
Total	107.941	24.907	17.442

Sites of specimen collection for prenatal screening were variable. In detail, 66.4% of specimens in Northern Italy, 61.2% in Central Italy and 43.3% in Southern Italy were collected from both lower vagina and rectum, as recommended by CDC guidelines.

Ninety-per-cent of respondents reported using Stuart or Amies transport medium for specimen transport, according to CDC guidelines, and 58% followed CDC indications for specimen processing (inoculation of specimen into selective broth and then subculture of incubated broth to an appropriate agar plate with or without initial step of direct inoculation to agar plate). Even if the total of respondents used appropriate agar plates for subculture of incubated broth only 12% used selective broths recommended by the CDC, e.g. Lim broth or Trans Vag broth. Eighty-eight percent of respondents reported a 24–48 h time interval for a positive response.

When asked about antibiotic susceptibility of GBS isolates, 4 centers did not performed susceptibility tests and 37 centers (36.6% of respondents) did not provide any information. Of the 60 centers where susceptibility testing was performed, 2 centers did not provide any resistance rates and in the remaining 58 centers resistance rates varied widely from less to 10 to more than 50% of strains with the majority of macrolide and clindamycin resistance reported to be between 10 and 30% of strain (33 and 29% of isolates were reported resistant to macrolide and clindamycin respectively).

## Discussion

We report here the results of the first nationwide survey aiming to describe the preventive practices for GBS-EOS in Italy both from the clinical and the microbiological perspectives.

Area-based information has been recently reported from two Italian regions [9–11], but no nationwide data on GBS prevention practices has been previously published. Although partial and not area-based, the information obtained through the current study is a preliminary step for planning future interventions.

### Obsteric and microbiologic practices

Almost all respondent units reported using a screening-based approach with culture collection at 35–37 weeks’ gestation. The culture-based strategy is recommended in the United States and in some European countries including Spain, Germany, Italy and France. The risk-based strategy is adopted in the United Kingdom, the Netherlands, Norway, and Denmark [12, 13] and reflects the view that the incidence of GBS-EOS will not reduce any further with the introduction of universal screening for GBS in pregnancy. No units routinely used bedside rapid molecular tests for intrapartum screening of pregnant women. Most units reported collecting vagino-rectal specimens (as recommended by CDC guidelines) [5]. However, only 58% of microbiological laboratories reported specimen processing consistent with CDC guidelines [5]. Furthermore, most units reported administering ampicillin (instead of penicillin) as their first line agent for IAP. Penicillin is recommended in the US guidelines [4, 5] because of its narrow spectrum, but, unfortunately, it is not produced in Italy. In case of allergic mothers without anaphylaxis, most units, reported administering clindamycin or, different from CDC guidelines, macrolides. Only few units reported using cefazolin (the agent recommended by CDC guidelines for pregnant women with low risks of anaphylaxis), or reported testing of antimicrobial susceptibility for allergic women. Increasing resistance of GBS to macrolides and clindamycin is a major concern worldwide, and the use of clindamycin is specifically discouraged by the CDC guidelines, unless the pregnant woman is at high risk of anaphylaxis and susceptibility test has been performed [5]. Furthermore, erythromycin is no longer recommended. Although most units reported administering antimicrobials every 4 h, some gave *intrapartum* antimicrobials every 6 h, a practice not provided by consensus guidelines.

### Neonatal practices

Despite the absence of national guidelines, all respondent units reported to have protocols to prevent GBS-EOS, in most cases consistent with CDC recommendations [5]. Although there was overall consensus for the majority of risk factors (the same as suggested by CDC guidelines) [5], some differences among centers were apparent. Some units reported collecting swabs at neonatal mucous sites (ear and throat). Based on CDC guidelines and the available literature, this practice should be discouraged, as most colonized neonates remain healthy. Most units reported not obtaining a sepsis workup for asymptomatic full-term neonates born to a GBS colonized mother exposed to adequate IAP. However, only ~25% of units reported observing asymptomatic neonates (without further testing or antimicrobial treatments) after exposure to inadequate IAP (as suggested by CDC guidelines) [5, 14, 15]. Indeed, approximately half of the units reported obtaining sepsis workup in full-term neonates and 30% reported administering empirical antimicrobial treatments; 43% reported antimicrobial treatment in preterm neonates, practices not consistent with CDC algorithms. Of note, only 60% of respondents would treat babies whose mothers had chorioamnionitis (as suggested by CDC guidelines). Algorithms for the management of neonates at risk for GBS-EOS, incorporated into the CDC and AAP guidelines have been repeatedly updated in recent years [5, 14, 15]. However, laboratory tests add little diagnostic information because of poor specificity and low positive predictive value, and their utility for a timely diagnosis of EOS in asymptomatic, high-risk neonates, has been questioned [15–19]. In order to reduce unnecessary evaluations and antimicrobial therapies, some recent guidelines currently recommend observation without further testing or treatments for asymptomatic full-term neonates born to a GBS colonized mother exposed to inadequate IAP [5, 15, 20, 21]. The lack of clinical evidence and uncertainty regarding the management of at risk neonates (mostly those who are asymptomatic), and the absence of national expert consensus guidelines have led in the current study to approaches which rely on local protocols or on the choice of individual clinicians. This can explain the differences between data here reported and CDC guidelines.

Although we have no information on the total number of blood and CSF cultures collected in neonates, in the current study rates of positive blood cultures were significantly higher in Central compared to Northern and Southern Italy. Furthermore, incidence rates of GBS-EOS in all geographical areas were higher than rates found in two recent prospective Italian area-based studies (0.10/1000 live births in Friuli Venezia Giulia, and 0.19/1000 live births in Emilia-Romagna.) [9, 10, 22]. This discrepancy may be due to the retrospective nature of the current study, as well as to inaccurate reporting; indeed, this study was not specifically designed to determine the incidence of EOS and no strict criteria for diagnosing infections were required.

Our study has some limitations. First, due to the low rate of feedback from clinicians and microbiological units, a selection bias may have occurred. Consequently, our data may only partially reflect the nationwide policies. Second, the study was conducted in 2012. This aspect is particularly important for microbiological tests, that may have been updated with newer available technologies since then. Finally, the estimate of positive blood and CSF cultures may have been biased and further, prospective, more accurate data are required to address this question. Nevertheless, this survey would be useful to give a cross-section of current practices for preventing GBS-EOS and prompt the publication of national guidelines.

## Conclusions

Our data show that the screening-based strategy is largely prevalent and, despite shortcomings and variation among centers, most protocols for preventing GBS EOS are consistent with CDC guidelines. Discrepancies in practices among prenatal and pediatric providers, and laboratorians may reflect the lack of guidance issued by public health organizations. The findings of our survey warrant the issue of new Italian consensus guidelines. Furthermore, in order to track the incidence of GBS-EOS and the impact of perinatal prevention interventions regionals data and laboratory-based surveillance systems are required.

## Additional file

Additional file 1: Table S1. Respondents divided by region and geographic area. (DOCX 96 kb)

## Abbreviations

AMCLI: Italian Association of Clinical Microbiologists
CSF: Cerebrospinal fluid
EOS: Early-onset sepsis
GBS: Group B Streptococcus
IAP: Intrapartum antibiotic prophylaxis
SIGO: Italian Society of Obstetricians and Gynecologists
SIN: Italian Society of Neonatology
