# Supplementary material for: Strategies for preventing group B streptococcal infections in newborns: a nation-wide survey of Italian policies
Source: Ital J Pediatr. 2017 Nov 2;43:98. doi: 10.1186/s13052-017-0409-1 (PMC5667472; doi:10.1186/s13052-017-0409-1)
Supplement: Additional file 1: Table S1. — Respondents divided by region and geographic area. (DOCX 96 kb) [file 13052_2017_409_MOESM1_ESM.docx]

**Supplemetary table**. Respondents divided by region and geographic area

| **Regions** | **Maternity units (n)** |  |  | **Microbiological**  **laboratories (n)** |  |
| --- | --- | --- | --- | --- | --- |
|  |  | **Neonatologists n (%)** | **Obstetricians**  **n (%)** |  | **Microbiologists**  **n (%)** |
| **Emilia Romagna** | 25 | 6 | 2 | 18 | 7 |
| **Friuli** | 10 | 3 | 1 | 11 | 6 |
| **Liguria** | 12 | 5 | 1 | 10 | 2 |
| **Lombardia** | 69 | 33 | 5 | 45 | 24 |
| **Piemonte** | 30 | 10 | 3 | 35 | 15 |
| **Trentino Alto Adige** | 13 | 1 | 3 | 7 | 3 |
| **Valle d’Aosta** | 1 | 0 | 0 | 1 | 0 |
| **Veneto** | 33 | 5 | 3 | 24 | 11 |
| **NORTH ITALY** | 193 | 63 (32.64) | 18 (9.3) | 151 | 68 (45) |
|  |  |  |  |  |  |
| **Lazio** | 42 | 6 | 1 | 24 | 6 |
| **Marche** | 15 | 4 | 0 | 16 | 6 |
| **Toscana** | 31 | 4 | 3 | 19 | 3 |
| **Umbria** | 13 | 1 | 0 | 11 | 2 |
| **CENTER ITALY** | 101 | 15 (14.8) | 4 (3.9) | 70 | 17 (24.3) |
|  |  |  |  |  |  |
| **Abruzzo** | 13 | 3 | 1 | 10 | 0 |
| **Basilicata** | 7 | 2 | 2 | 5 | 1 |
| **Calabria** | 19 | 4 | 0 | 13 | 4 |
| **Campania** | 46 | 7 | 2 | 24 | 1 |
| **Molise** | 5 | 0 | 0 | 2 | 1 |
| **Puglia** | 40 | 7 | 2 | 32 | 3 |
| **Sardegna** | 12 | 2 | 0 | 12 | 2 |
| **Sicilia** | 57 | 4 | 5 | 19 | 4 |
| **SOUTH ITALY** | 199 | 29 (14.6) | 12 (6) | 117 | 16 (13.7) |
|  |  |  |  |  |  |
| **TOTAL** | 493 | 107 (21.7) | 34 (6.9) | 338 | 101 (29.8) |
